# miR-187/PDLIM1 Gets Involved in Gastric Cancer Progression and Cisplatin Sensitivity of Cisplatin by Mediating the Hippo-YAP Signaling Pathway

**DOI:** 10.1155/2022/5456016

**Published:** 2022-09-17

**Authors:** Yeru Tan, Yuehua Li, Hongbo Zhu, Xiaoping Wu, Kai Mei, Pian Li, Qiao Yang

**Affiliations:** ^1^The First Affiliated Hospital, Department of Medical Oncology, Hengyang Medical School, University of South China, Hengyang, Hunan 421001, China; ^2^The First Affiliated Hospital, Department of Oncology Radiotherapy, Hengyang Medical School, University of South China, Hengyang, Hunan 421001, China

## Abstract

Gastric cancer (GC) is one of the most prevalent malignancies in the digestive system across the world. The function and mechanism of PDLIM1, a cancer-suppressing gene, in gastric cancer progression remain unclear. This study is aimed at investigating the expression features and function of PDLIM1 in GC. RT-qPCR and western blot were used to compare the profiles of PDLIM1 and miR-187 between GC and normal tissues. The cell models of PDLIM1 overexpression and low expression were established in gastric cancer cell lines MKN45 and AGS. CCK8 and BrdU assays measured cell proliferation. Flow cytometry monitored cell apoptosis. Transwell analyzed cell invasion and migration. The influence of miR-187 overexpression on gastric cancer development was assessed. We predicted the targeted correlation between miR-187 and PDLIM1 through bioinformatics, which was corroborated via dual luciferase activity assay and RIP. Meanwhile, the cell model of PDLIM1 overexpression was built in AGS cells transfected with miR-187 mimics. A rescue experiment was conducted to assess the impact of PDLIM1 overexpression on the procancer function of miR-187. As a result, in contrast with normal paracancer tissues, PDLIM1 was substantially downregulated in GC tissues. Moreover, PDLIM1 overexpression considerably dampened proliferation, invasion, and migration in GC cells, boosted the cell apoptosis, and bolstered their sensitivity to cisplatin. PDLIM1 knockdown or miR-187 overexpression dramatically fostered GC cell proliferation, invasion, and migration and repressed cell apoptosis. Mechanism studies demonstrated that PDLIM1 vigorously restrained the profiles of the Hippo-YAP signaling pathway and the downstream target genes. miR-187 targeted PDLIM1, while miR-187 overexpression cramped PDLIM1 expression. The rescue experiment suggested that PDLIM1 overexpression weakened the procancer function of miR-187 in GC cells. In conclusion, our study demonstrated that PDLIM1 presented a low expression in GC tissues, while miR-187/PDLIM1 participated in GC development and cisplatin sensitivity by mediating the Hippo-YAP signaling pathway.

## 1. Introduction

The incidence rate of gastrointestinal tumors is increasing in recent years [1]. Gastric cancer (GC), one of the most prevailing malignancies in the digestive system across the world, is also the fifth biggest contributor to cancer and the third biggest cause of cancer-related death worldwide [2, 3]. It mainly arises from helicobacter pylori infection, age, high salt intake, and diets deficient in fruits and vegetables [4]. Existing treatment strategies for GC includes genome classification, surgical resection and treatment, systemic radiotherapy, and chemotherapy as well as targeted therapy and immunotherapy. The onset of GC is not easy to be found, and the recurrence and metastasis rate after surgery is high. Therefore, there are still many difficulties in its treatment [5, 6]. Cisplatin (DDP), an efficacious chemotherapy drug, is of great value in treating various cancers like GC [7]. However, GC patients often experience relapse and metastasis due to resistance to DDP therapy, with limited therapeutic effect [8]. Thus, the exact mechanism that influences GC progression and DDP resistance needs to be clarified. This is pivotal to exploring efficacious targets for GC treatment.

MicroRNAs (miRNAs), small noncoding RNAs about 18-25 nucleotides in length, can combine with the 3'-untranslated region (3'UTR) of target mRNAs to suppress the profiles of target genes [9, 10]. miRNAs can be used as prognostic biomarkers. They can exert a crucial function in modulating many biological processes of multiple human diseases, such as cancer [11]. miR-187, a member of the miRNA family, partakes in the development of umpteen cancers, including nonsmall cell lung cancer [12] and cervical cancer [13]. More of note, miR-187 can serve as a new serum biomarker for the early detection of GC [14]. Furthermore, miR-187 acts as a tumor suppressor in the context of GC, which hints that it may be a biomarker and therapeutic target for GC patients [15]. Nevertheless, the exact mechanism of miR-187 influencing GC development needs to be improved.

PDLIM1 (PDZ and LIM domain protein 1), also called CLP36, Elfin, or CLIM1, reportedly interacts with many proteins, including *ɑ*-actinin, paladin, FHL1, and EGFR, thus playing a significant part in cytoskeletal tissues, neuronal signals, and organ development [16]. Increasing evidence has indicated that PDLIM1 expression is dysregulated in many tumors like glioma [17] and breast cancer [18]. Additionally, the anti-PDLIM1 auto-antibody can serve as a novel serum marker for ovarian cancer [19], but its function in GC development has been rarely reported.

The Hippo (Hpo) pathway was first identified in Drosophila. It has been reported to be an evolutionarily conserved signaling pathway that modulates cell proliferation, tissue homeostasis, and tissue regeneration [20, 21]. Reportedly, dysregulation of the pathway pertains to abnormal tissue growth and tumorigenesis. In the mammalian system, the relevant phosphorylated protein (YAP) is replaced in the cytoplasm to promote degradation when the Hippo signaling pathway is initiated. Inactivation of the Hippo pathway moves unphosphorylated YAP into the nucleus, hence eliciting the transcriptional activities of genes associated with cell growth [22, 23]. The Hippo pathway has a huge function in different cancers, but its correlation with PDLIM1 has not been elucidated. Thus, confirming the correlation between PDLIM1 and the regulation of the Hippo pathway seems to be of great significance in human cancers, particularly GC.

In this study, we investigated the function and exact mechanism of PDLIM1 during GC progression. The miR-187/PDLIM1 is involved in GC development and cisplatin sensitivity by regulating the Hippo-YAP signaling pathway. This provides a new idea for the clinical treatment of GC and its resistance to DDP.

## 2. Materials and Methods

### 2.1. Clinical Specimen Collection and Processing

The cancer tissues and normal paracancer tissues of 45 GC patients who received gastrectomy from October 2015 to March 2016 in our hospital were harvested. Prior to the surgery, they were not subjected to chemotherapy, radiotherapy, or other adjuvant therapies. The specimens in the control group were taken from the paracancer tissues of the same patients (at least 3 cm away from the surgical margin). No cancer tissues were detected during pathological examination following the surgery. In accordance with the standards of the World Health Organization (WHO), we substantiated the diagnosis of GC from the perspective of pathology. All specimens were kept in -196°C liquid nitrogen in preparation for RNA extraction.

### 2.2. Cell Culture and Transfection

Human GC cell lines (MKN-45, AGS) and human DDP-resistant GC cell lines (AGS/DDP) were obtained from American Type Culture Collection (ATCC; Manassas, Virginia, USA). The cells were grown in the PRMI-1640 medium supplemented with 10% FBS (Invitrogen, Carlsbad, CA, US) at 37°C with 5% CO_2_. The cells were passaged every two or three days.

The cells were inoculated into 6-well plates with a density of 5 × 10^6^/well. We transfected PDLIM1 overexpression plasmid (pcDNA3.1-PDLIM1), PDLIM1 low expression plasmid (sh-PDLIM1) and its corresponding negative control (sh-NC), and miR-187 mimics and their corresponding negative control fragment (miR-NC) into MKN-45 and AGS cells according to the instructions of FuGENE®HD Transfection Reagent (Roche, Shanghai, China), respectively. Cells in each group were incubated in an incubator with 5% CO_2_ at 37°C. 24 hours posttransfection, the steadily growing cells in each group were harvested.

### 2.3. Cell Counting Kit-8 (CCK8) Assay

MKN45 and AGS cells were seeded into 96-well plates with a density of 1 × 10^3^ cells/well and incubated for 24 hours. Subsequently, 10 *μ*l of CCK8 reagent (Dojindo Molecular Technologies, Kumamoto, Japan) was added into each well. After the cells were incubated for an hour at 37°C, the OD450 value of each well was determined by a spectrophotometer (Bio-Rad, CA, USA).

### 2.4. DDP Sensitivity Detection

We measured the viability of DDP-resistant GC cells under the impact of different DDP concentrations using 3-(4,5-dimethyl-2-thiazolyl)-2,5-diphenyl-2H-tetrazolium bromide (MTT; Sigma-Aldrich) with a view to confirming and visualizing the semi-inhibitory concentration (IC50) value of DDP. The IC50 value was defined as the concentration of DDP corresponding to 50% of cell viability inhibition rates in the curve [24].

### 2.5. Bromodeoxyuridine (BrdU) Staining

Following transfection, GC cells in each group were moved onto coverslips (Beyotime, Shanghai, China) for 12 hours of culture. Later, the cells were incubated with BrdU solution (Beyotime, Shanghai, China) for 6 hours. The culture medium was discarded. GC cells were immobilized with 4% paraformaldehyde for 30 minutes, incubated with anti-BrdU antibody (Beyotime, Shanghai, China) for an hour at room temperature (RT), and flushed with phosphate buffer saline (PBS). The number of positive BrdU cells was calculated [25].

### 2.6. Transwell Assay

Transwell examined cell invasion and migration. The cells posttransfection were harvested. With the cell density set to 4 × 10^4^, the cells were suspended in a serum-free medium supplemented with 1 *μ*g/mL mitomycin C. Then, the cells were inoculated into an upper compartment precoated with Matrigel, while 10% fetal bovine serum (FBS) was given to the lower compartment. Subsequent to 24 hours of incubation at 37°C, substrates and cells that failed to pass the membrane surface in the upper compartment were wiped off. The cells were rinsed, immobilized with paraformaldehyde for 10 minutes, and dyed with 0.5% crystal violet. A microscope was adopted to observe cell invasion. As for the cell migration test, Matrigel was not administered to the upper Transwell chamber, but the other steps were similar to those in the invasion test.

### 2.7. Flow Cytometry

Annexin V-FITC double staining was implemented to measure cell apoptosis. Following 24 hours of transfection, the cells were digested with trypsin, harvested, and inoculated into 6-well plates for 24 hours of further culture, with the cell density adjusted to 2 × 10^6^ cells/well. The supernatant was removed. The cells were flushed with precooled PBS twice and resuspended with 1 × binding buffer. Later, 5 *μ*L Annexin V-FITC and 5 *μ*L PI were administered to the cell suspension, and they were conflated thoroughly. The cells were incubated at RT for 15 minutes, and the cell apoptosis rate was confirmed by a flow cytometry instrument within an hour. Apoptosis rate = number of apoptotic cells/(number of apoptotic cells + number of normal cells) × 100%. All procedures were done as instructed by the apoptosis kit (Yeasen Biotech Co., Ltd., Shanghai, China).

### 2.8. RT-qPCR

TRIzol reagent was utilized to extract total RNA from the cells. As per the supplier's instructions, the PrimeScript™ RT Reagent kit (Invitrogen, Shanghai, China) was taken to reverse-transcribe the RNA into cDNA. The Bio-Rad CFX96 quantitative PCR system and SYBR were utilized for qPCR in line with the supplier's stipulation. The conditions for PCR were as follows: 5 minutes of predenaturation at 95°C, 15 seconds of denaturation at 95°C, and 30 seconds of annealing at 60°C. *β*-Actin was adopted as the internal parameter for confirming PDLIM1 and miR-187 expressions. The 2^−ΔΔCt^ approach was adopted for statistical analysis. Each experiment was duplicated three times. Guangzhou Ruibo Company took on the design and synthesis of the primers. The primer sequences are detailed in Table 1.

### 2.9. Western Blot

The cellular protein was isolated with protein lysis buffer (Roche, USA). Subsequently, 50 *μ*g of the total protein was subjected to SDS-PAGE and transferred onto polyvinylidene fluoride (PVDF) membranes. After blocked with 5% skimmed milk in PBST for an hour, the membranes were washed with TBST three times, and incubated with primary antibodies including Anti-Bax antibody (ab32503, 1 : 1000, Abcam, USA), Anti-Bcl-2 antibody (ab32124, 1 : 1000, Abcam, USA), Anti-Bad antibody (ab32445, 1 : 1000, Abcam, USA), Anti-PDLIM1 antibody (ab129015, 1 : 1000, Abcam, USA), Anti-Amphiregulin antibody (ab89119, 1 : 1000, Abcam, USA), Anti-Myc antibody (ab185656, 1 : 1000, Abcam, USA), Anti-CCND2 antibody (ab267318, 1 : 1000, Abcam, USA), Anti-YAP antibody (ab52771, 1 : 1000, Abcam, USA), Anti-p-YAP antibody (ab254343, 1 : 1000, Abcam, USA), Anti-*β*-actin antibody (ab115777, 1 : 1000, Abcam, USA), and Anti-Lamin A antibody (ab108595, 1 : 1000, Abcam, USA) at 4°C overnight. The membranes were washed with TBST and then incubated with the horseradish peroxidase (HRP)-labeled anti-rabbit secondary antibody (1 : 3000, Abcam, USA) for an hour at RT. The bands were developed with Pierce™ ECL Western Blotting Substrate (Invitrogen, USA). The gray value of each protein was analyzed using Image J analysis software (National Institutes of Health, USA).

### 2.10. Dual Luciferase Reporter Assay

We predicted the target genes of miR-187 through TargetScan. Pmir-GLO-NC, pmir-GLO-PDLIM1-wt, pmir-GLO-PDLIM1-mut, miR-NC, and miR-187, ordered from Sangon Biotech (Shanghai, China), were transfected into the cells with Lipofectamine™2000 (Invitrogen, Carlsbad, CA, USA). Subsequent to incubation, the cells were harvested and flushed with PBS twice. The Dual-Lucy Assay Kit (Progema, Madison, WI, USA) was used for the assay [26].

### 2.11. RIP Assay

To further confirm the correlation between miR-187 and PDLIM1, we utilized the Magna RIP™ RNA Binding Protein Immunoprecipitation Kit (Millipore, Bedford, MA, USA) for RIP analysis. Then, AGS cells (2 × 10^7^) transfected with miR-187 or its negative control were harvested and subjected to 200 *μ*L of RIP lysis buffer. They were lysed on ice for 5 minutes and centrifuged at 1500 rpm for 15 minutes to produce the supernatant. The extracts were incubated with Anti-Ago2 or Anti-IgG (Sigma) overnight. Subsequently, magnet beads were flushed with a washing buffer five times, and the supernatant was removed. The beads were lysed with the protease K lysate at 55°C for 30 minutes. The supernatant was put in a new centrifuge tube. The total RNA was extracted through phenol–chloroform-isoamyl alcohol extraction and purified via isopropanol centrifugation. The coprecipitated RNA was isolated and analyzed through RT-qPCR.

### 2.12. Statistical Analysis

GraphPad Prism 8 was used for statistical analysis. Experimental data were presented as mean ± standard deviation (SD). One-way ANOVA and Student's *t*-test were taken for comparison. Two-sided *P* < 0.05 was regarded as statistically significant. The experiment was duplicated three times.

## 3. Results

### 3.1. PDLIM1 Expression Is Lowered in GC Tissues and Cell Lines

**Figure 1:**
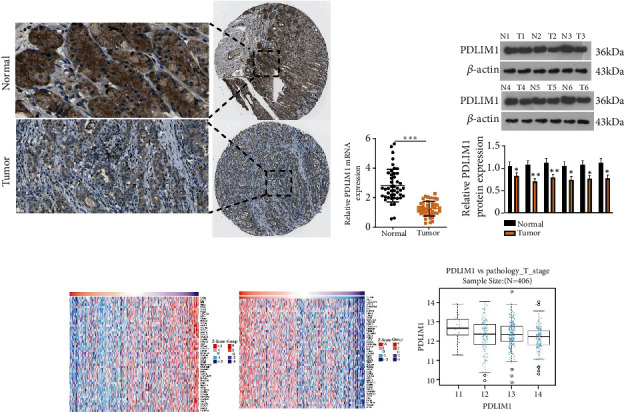
PDLIM1 expression is lowered in GC tissues and cell lines. (a) The database (https://www.proteinatlas.org/) was introduced to examine the positive profile of PDLIM1 in GC tissues. (b, c) RT-PCR and western blot evaluated the profile of PDLIM1 in GC tissues and normal paracancer tissues. (d) The coexpressed genes of PDLIM1 in GC (LindOmics). The heat maps exhibited the positively and negatively coexpressed Top-50 genes of PDLIM1 in GC. (e) The staging of tumors. ^∗∗∗^*P* < 0.001 (vs. the Normal group). *N* = 45.

To determine whether PDLIM1 could exert a significant function in GC development, we uncovered that PDLIM1 expression was remarkably attenuated in GC tissues (vis-a-vis normal paracancer tissues) through the database (https://www.proteinatlas.org/) (Figure 1(a)). RT-PCR (*P* < 0.05, Figure 1(b)) and western blot (Figure 1(c)) confirmed that the profile of PDLIM1 was lowered in GC tissues in contrast with normal paracancer tissues. Additionally, the coexpressed genes of PDLIM1 in the context of GC were analyzed through the LinkedOmics database (Figure 1(d)). They were negatively correlated with the staging of tumors (Figure 1(e)). The correlation between PDLIM1 expression and clinical characteristics in the tissue specimens of GC patients was shown in Table 2. These discoveries revealed that PDLIM1 pertained to the malignant phenotype of GC cells and possibly played a procancer role.

### 3.2. PDLIM1 Hampers GC Cell Proliferation, Migration, and Invasion and Boosts Apoptosis

**Figure 2:**
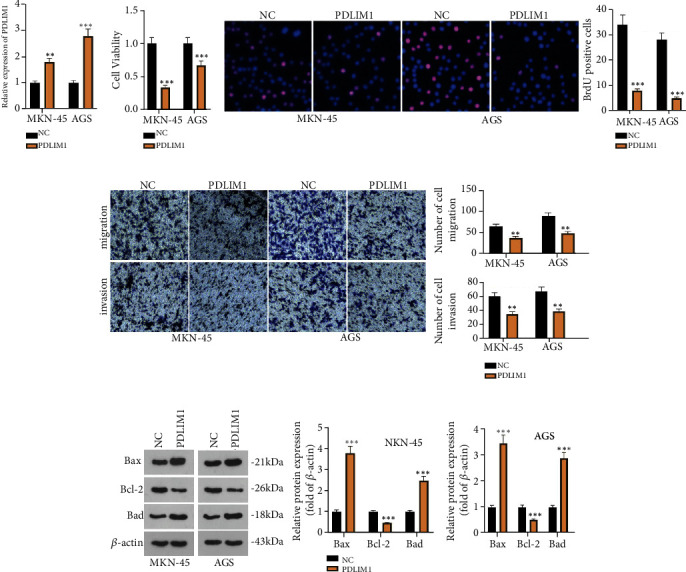
PDLIM1 hampers GC cell proliferation, migration, and invasion and boosts apoptosis. A cell model of PDLIM1 overexpression was built in MKN-45 and AGS cells. (a) RT-qPCR confirmed the profile of PDLIM1. (b, c) CCK8 and BrdU verified the viability of MKN-45 and AGS cells. (d) Transwell detected GC cell invasion and migration. (e) Flow cytometry examined cell apoptosis. ^∗∗^*P* < 0.01, ^∗∗∗^*P* < 0.001 (vs. the NC group). *N* = 3.

To confirm the impact of PDLIM1 on GC development, we established a cell model of PDLIM1 overexpression in MKN-45 and AGS (*P* < 0.05, Figure 2(a)). CCK8 and BrdU denoted that PDLIM1 overexpression brought about a substantial reduction in cell proliferation (*P* < 0.05, Figures 2(b) and 2(c)). Transwell indicated that PDLIM1 overexpression vigorously lessened cell invasion and migration (*P* < 0.05, Figure 2(d)). Flow cytometry revealed that cell apoptosis was remarkably augmented in the PDLIM1 group vis-a-vis the control group (*P* < 0.05, Figure 2(e)). Western blot suggested that PDLIM1 overexpression notably attenuated the profile of the anti-apoptotic protein Bcl-2 and augmented the profiles of proapoptotic proteins Bax and Bad (*P* < 0.05, Figure 2(f)). These outcomes demonstrated that PDLIM1 overexpression weakened the malignant biological behaviors of GC cells.

### 3.3. PDLIM1 Knockdown Bolsters GC Cell Proliferation, Migration, and Invasion and Hinders Apoptosis

**Figure 3:**
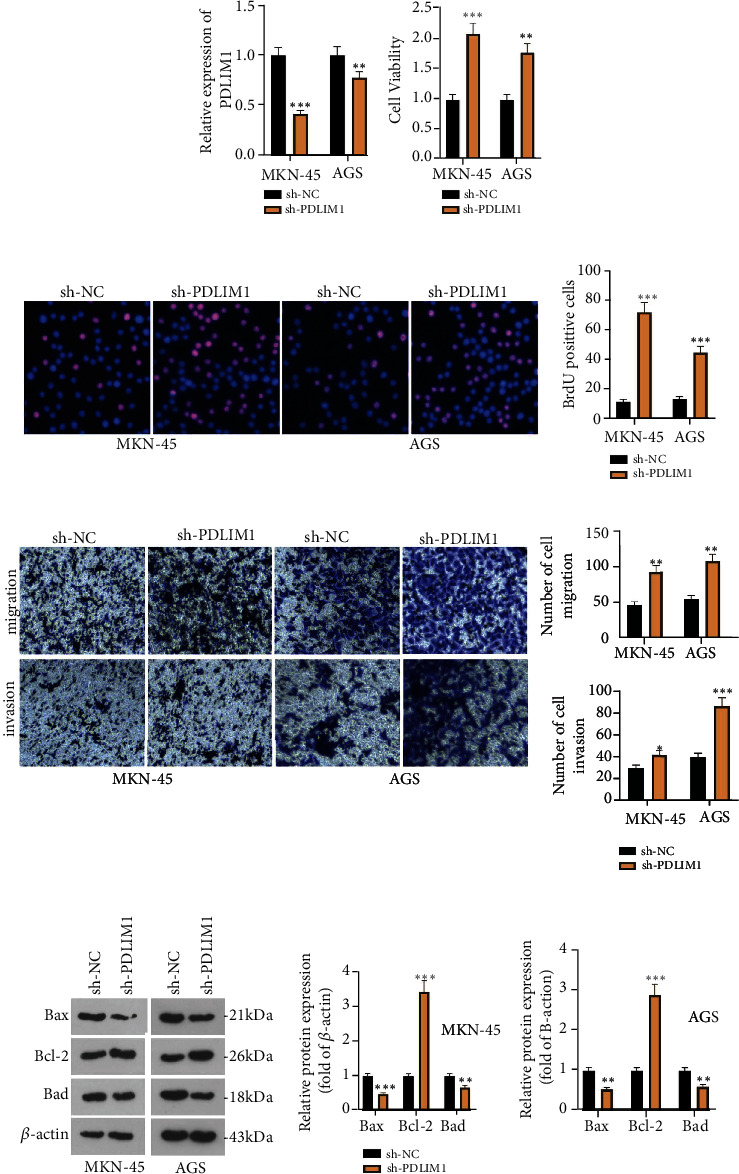
PDLIM1 knockdown bolsters GC cell proliferation, migration, and invasion and suppresses apoptosis. A cell model of PDLIM1 knockdown was established in MKN-45 and AGS cells. (a) RT-PCR determined PDLIM1 expression. (b, c) CCK8 and BrdU examined the viability of MKN-45 and AGS cells. (d) Transwell monitored cell invasion and migration. (e) Flow cytometry measured cell apoptosis. ^∗∗^*P* < 0.01, ^∗∗∗^*P* < 0.001 (vs. the sh-NC group). *N* = 3.

To confirm the influence of PDLIM1 on GC development, we built a PDLIM1 knockdown model in MKN-45 and AGS cells (*P* < 0.05, Figure 3(a)). CCK8 and BrdU denoted that PDLIM1 knockdown considerably augmented cell viability (*P* < 0.05, Figures 3(b) and 3(c)). Transwell indicated that PDLIM1 knockdown greatly strengthened cell invasion and migration (*P* < 0.05, Figure 3(d)). Flow cytometry unraveled that by contrast to the control group, the PDLIM1 group had a distinct decline in cell apoptosis (*P* < 0.05, Figure 3(e)). Western blot suggested that PDLIM1 knockdown dramatically heightened Bcl2 expression and lowered Bax and Bad expressions (*P* < 0.05, Figure 3(f)). These findings demonstrated that PDLIM1 inhibition boosted the malignant biological behaviors of GC cells.

### 3.4. PDLIM1 Overexpression Strengthens the Sensitivity of GC Cells to Cisplatin

**Figure 4:**
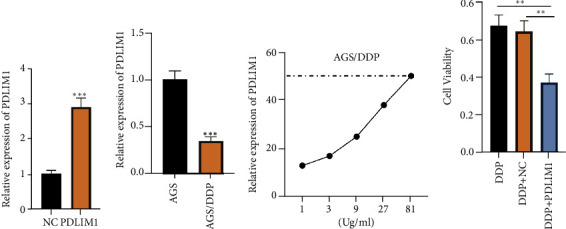
PDLIM1 overexpression strengthens the sensitivity of GC cells to cisplatin. (a) PDLIM1 or NC was transfected into AGS/DDP cells resistant to DDP. The profile of PDLIM1 was determined via RT-qPCR. (b) RT-qPCR confirmed the mRNA profile of PDLIM1 in the two cell lines. (c) The IC50 value to DDP in the cell lines was determined by CCK8. (d) The DDP-resistant cells were transfected with PDLIM1 and then treated with 1 *μ*g/mL DDP. CCK8 monitored cell proliferation. The apoptosis rate of each group was calculated. ^∗∗^*P* < 0.01, ^∗∗∗^*P* < 0.001 vs. the DDP group). *N* = 3.

To better understand the function of PDLIM1 overexpression in modulating DDP-resistance in AGS/DDP cells, we synthesized PDLIM1 or NC. First, they were transfected into AGS/DDP cells. PDLIM1 expression was confirmed via RT-qPCR. It turned out that the profile of PDLIM1 was notably higher than that of NC (*P* < 0.05, Figure 4(a)). To verify whether PDLIM1 presented different expressions in the two cell lines, we performed RT-qPCR for analysis. As a result, the mRNA level of PDLIM1 was evidently lower in AGS/DDP than in AGS (*P* < 0.05, Figure 4(b)). Moreover, CCK8 was performed to confirm the IC50 value, thus testing the resistance of AGS/DDP to DDP. The outcomes suggested that the IC50 value to DDP in AGS/DDP was 81.3 *μ*g/mL (*P* < 0.05, Figure 4(c)). Later, AGS/DDP cells, treated with DDP, were transfected with PDLIM1 or NC. CCK8 examined cell viability. PDLIM1 overexpression culminated in a notably lower survival rate in contrast with the DDP group (*P* < 0.05, Figure 4(d)). These findings confirmed that an increase in PDLIM1 markedly bolstered AGS/DDP cell apoptosis, whereas PDLIM1 overexpression vigorously attenuated the resistance of AGS/DDP cells to DDP.

### 3.5. miR-187 Overexpression Facilitates GC Progression

**Figure 5:**
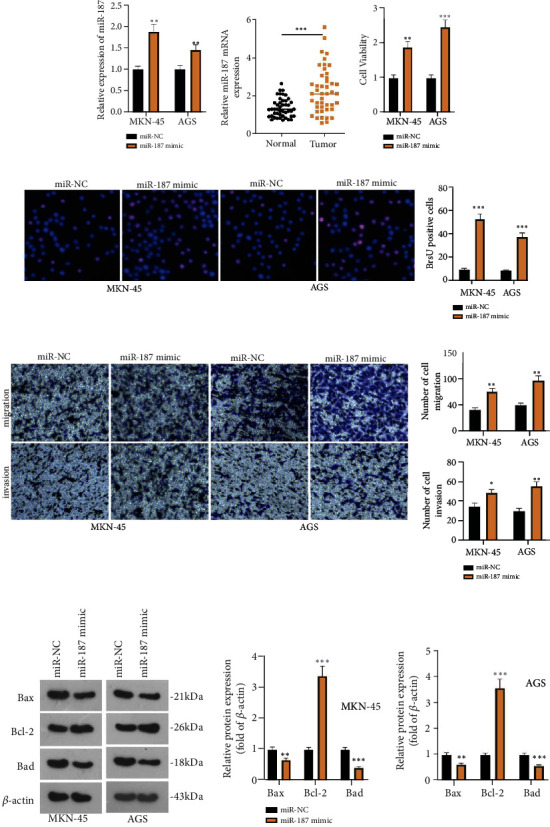
miR-187 overexpression facilitates GC development. A cell model of miR-187 overexpression was established in MKN-45 and AGS cells. (a) RT-qPCR examined miR-187 expression. (b) RT-qPCR confirmed miR-187 expression in GC tissues and normal paracancer tissues. (c, d) CCK8 and BrdU measured MKN-45 and AGS cell viability. (e) Transwell monitored invasion and migration in MKN-45 and AGS cells. (f) Flow cytometry evaluated cell apoptosis. (g) Western blot verified the profiles of Bcl2, Bax, and Bad. ^∗∗^*P* < 0.01, ^∗∗∗^*P* < 0.001 (vs. the miR-NC group). *N* = 3.

To determine the influence of miR-187 on GC development, we established a cell model of miR-187 overexpression in MKN-45 and AGS cells (*P* < 0.05, Figure 5(a)). RT-PCR exhibited that the profile of miR-187 was greatly heightened in GC tissues as compared with normal paracancer tissues (*P* < 0.05, Figure 5(b)). CCK8 and BrdU indicated that overexpression of miR-187 contributed to a conspicuous increase in cell proliferation (*P* < 0.05, Figures 5(c) and 5(d)). Transwell denoted that overexpression of miR-187 dramatically strengthened cell invasion and migration (*P* < 0.05, Figure 5(e)). Flow cytometry revealed that as opposed to the control group, the miR-187 group had a considerable reduction in cell apoptosis (*P* < 0.05, Figure 5(f)). These discoveries demonstrated that the transfection of miR-187 mimics stepped up the malignant development of GC cells.

### 3.6. miR-187 Targets PDLIM1

**Figure 6:**
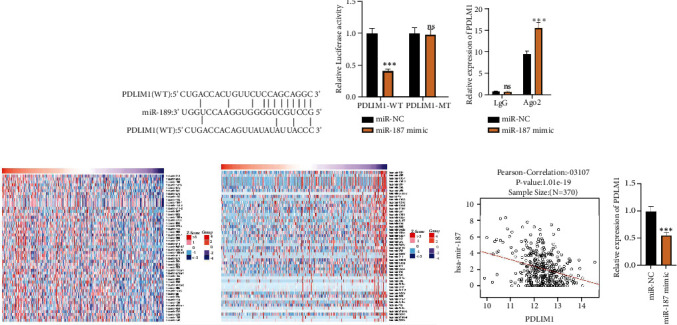
miR-187 targets PDLIM1. (a) The Starbase database (http://starbase.sysu.edu.cn/) forecast the binding target of PDLIM1 and miR-187. (b, c) Dual luciferase activity assay and RIP corroborated the binding relationship between PDLIM1 and miR-187. (d) The coexpressed genes of miR-187 in GC (LindOmics). The heat maps exhibited the positively and negatively coexpressed Top-50 genes of miR-187 in GC. (e) PDLIM1 was negatively correlated with miR-187 in GC. (f) RT-qPCR confirmed PDLIM1 expression in MKN-45 cells. ns *P* > 0.05, ^∗∗∗^*P* < 0.001. *N* = 3.

By using the Starbase database (http://starbase.sysu.edu.cn/), we predicted that PDLIM1 was the target of miR-187 (Figure 6(a)). To better understand the targeted correlation between miR-187 and PDLIM1, we implemented dual luciferase activity assay. It transpired that miR-187 vigorously suppressed PDLIM1-WT activity (*P* < 0.05, Figure 6(b)) but exerted little impact on PDLIM1-MUT (*P* < 0.05, Figure 6(b)). RIP revealed that following miR-187 transfection, PDLIM1 precipitated in the Ago2 antibody group was more than that in the IgG group. This hinted that PDLIM1 combined with Ago2 protein via miR-187 (*P* < 0.05, Figure 6(c)). To probe the potential mechanism of miR-187, we evaluated the coexpressed genes of miR-187 in the context of GC via the LinkedOmics database. As a result, PDLIM1 was negatively associated with miR-187 in GC (Figures 6(d) and 6(e)). Later, the profile of PDLIM1 subsequent to miR-187 overexpression in MKN-45 cells was measured. By contrast to the control group, miR-187 overexpression vigorously repressed PDLIM1 expression (*P* < 0.05, Figure 6(f)). These phenomena demonstrated that miR-187 targeted and negatively modulated PDLIM1 expression in GC cells.

### 3.7. PDLIM1 Influences the Hippo-YAP Pathway

**Figure 7:**
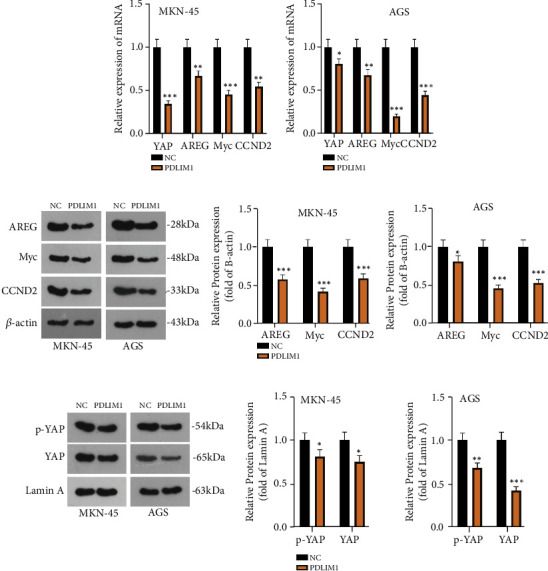
PDLIM1 influences the Hippo-YAP pathway. (a, b) RT-qPCR confirmed the profiles of YAP and its target genes in the cells. (c) Western blot verified the profiles of YAP and its target genes in the cells. (d) Western blot measured the abundance of YAP and phosphorylated YAP in the cell nucleus. ^∗^*P* < 0.05, ^∗∗^*P* < 0.01, ^∗∗∗^*P* < 0.001 (vs. the NC group). *N* = 3.

The Hippo pathway exerted a significant function in cancer progression [27]. We delved into the regulatory impact of PDLIM1 on the Hippo-YAP pathway. In Figures 7(a) and 7(b), RT-qPCR denoted that the mRNA levels of YAP and its target genes AREG, Myc, and CCND2 were negatively modulated by PDLIM1 in MKN-45 and AGS cells (*P* < 0.05, Figures 7(a) and 7(b)). This outcome was also substantiated by western blot subsequently (*P* < 0.05, Figure 7(c)). As YAP was translocated into the nucleus and influenced the transcription of its target genes, we checked the abundance of nuclear and phosphorylated YAP in the nucleus.  Figure 7(d) displayed that the levels of YAP and phosphorylated YAP were lowered in the nuclei of cells with PDLIM1 overexpression (*P* < 0.05, Figure 7(d)). These discoveries unraveled that PDLIM1 impeded the Hippo/YAP signaling pathway.

### 3.8. PDLIM1 Overexpression Weakens the Procancer Function of miR-187 in GC Cells

**Figure 8:**
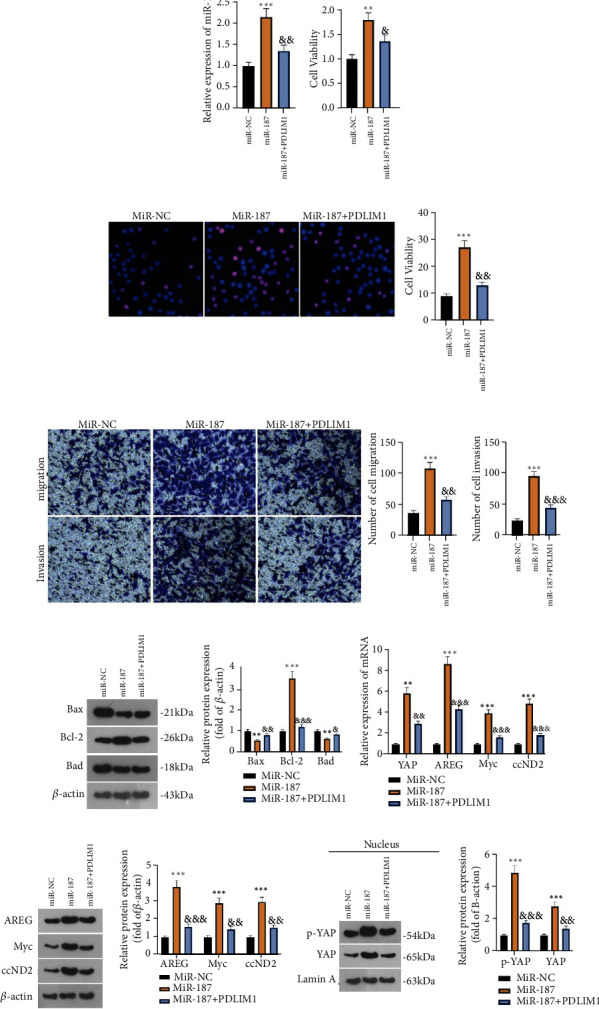
PDLIM1 overexpression weakens the procancer function of miR-187 in GC cells. AGS cells, already transfected with miR-187 mimics, were transfected with PDLIM1 overexpression plasmid. (a) RT-qPCR confirmed miR-187 expression. (b-c) CCK8 and BrdU staining measured cell proliferation. (d) Transwell monitored AGS cell migration and invasion. (e) Flow cytometry evaluated cell apoptosis. (f) Western blot determined the protein profiles of Bax, Bad, and Bcl2. (g) RT-qPCR verified the mRNA levels of YAP, AREG, Myc, and CCDN2. (h) Western blot tested the levels of AREG, Myc, and CCND2 as well as those of YAP and phosphorylated YAP in the nucleus. ^∗∗^*P* < 0.01, ^∗∗∗^*P* < 0.001 (vs. the miR-NC group). ^&^*P* < 0.05, ^&&^*P* < 0.05, ^&&&^*P* < 0.001 (vs. the miR-187 group). *N* = 3.

To dig deeper into the influence of PDLIM1 overexpression on the procancer function of miR-187 in GC cells, we transfected PDLIM1 overexpression plasmid into AGS cells already transfected with miR-187 mimics and confirmed the transfection efficiency via RT-qPCR. As a consequence, in contrast with the control group, miR-187 expression was notably heightened in the miR-187 group, whereas PDLIM1 lowered the profile of miR-187 (*P* < 0.05, Figure 8(a)). CCK8 and BrdU staining measured cell proliferation. As opposed to the control group, the miR-187 group went through a notable increase in cell proliferation. But in contrast with the miR-187 group, PDLIM1 overexpression vigorously dampened AGS cell proliferation (*P* < 0.05, Figures 8(b) and 8(c)). Transwell revealed that in contrast with the control group, the miR-187 group experienced a distinct increase in cell migration and invasion. But as compared with the miR-187 group, the miR − 187 + PDLIM1 group had a substantial decline in AGS cell migration and invasion (*P* < 0.05, Figure 8(d)). Flow cytometry confirmed that by contrast to the NC group, there was a reduction in AGS cell apoptosis in the miR-187 group. As compared with the miR-187 group, AGS cell apoptosis was augmented in the miR − 187 + PDLIM1 group (*P* < 0.05, Figure 8(e)). Western blot determined the profiles of Bax, Bad, and Bcl2. It turned out that the protein profiles of Bax and Bad were abated, and Bcl2 expression was elevated in the miR-187 group vis-a-vis the miR-NC group.

By contrast to the miR-187 group, Bax and Bad expressions were evidently heightened, while Bcl2 expression was lowered in the miR − 187 + PDLIM1 group (*P* < 0.05, Figure 8(f)). The mRNA levels of YAP, AREG, Myc, and CCND2 were dramatically downregulated in the miR − 187 + PDLIM1 group vis-a-vis the miR-187 group (*P* < 0.05, Figure 8(g)). This finding was also corroborated by western blot later (*P* < 0.05, Figure 8(h)). These phenomena demonstrated that PDLIM1 overexpression weakened the procancer function of miR-187, thus slowing GC progression.

## 4. Discussion

Recently, because of changes in dietary habits, the incidence rate of GC has been relatively lowered. Nevertheless, GC shows strong invasive and metastatic features, and its early detection is poor. Many GC patients have already entered into the advanced stage upon the first diagnosis, demonstrating fast progression and poor prognosis [28–30]. mRNAs have high specificity and are aberrantly expressed under different pathological and physiological circumstances. Therefore, they have drawn enormous attention as underlying diagnostic and predictive biomarkers these years [31, 32]. Here, we confirmed that PDLIM1 was a novel GC inhibitor. PDLIM1 overexpression vigorously hampered proliferation, invasion, and migration in GC cells and strengthened their sensitivity to cisplatin. These discoveries exhibited that PDLIM1 could be utilized as a novel target for GC treatment.

PDLIM1 exerts a significant function in umpteen cancers. For instance, PDLIM1 stabilizes *β*-catenin at the cell-to-cell junction to suppress epithelial mesenchymal transformation and metastatic potential in colorectal cancer [33]. Moreover, PDLIM1 promotes proliferation and impedes apoptosis to play a carcinogenic part in chronic myeloid leukemia (CML) [34]. PDLIM1, also called CLP36, has been identified as a tumor antigen that elicits antibody response [35]. These findings represent the diverse functions of PDLIM1 in multiple cancers. Notwithstanding, the function and mechanism of PDLIM1 in the context of GC remain obscure. Here, we discovered that PDLIM1 expression was lowered in GC tissues and cell lines; the function of PDLIM1 was inextricably associated with a lot of physiological parameters of GC, including proliferation, invasion, migration, and apoptosis. These statistics demonstrated that PDLIM1 overexpression repressed GC cell proliferation, invasion, and migration and bolstered apoptosis, whereas PDLIM1 inhibition facilitated the malignant development of GC cells.

Chemotherapy has been utilized to treat patients with unresectable gastric tumors with a view to reducing recurrence and metastasis [36]. Furthermore, perioperative chemotherapy can dramatically ameliorate the prognosis of patients with resectable tumors [37]. Nevertheless, 70%~90% of GC patients may relapse on account of chemotherapy resistance [38, 39]. It is still a big challenge in GC treatment. Further research on the mechanism of chemical resistance is in urgent need. Here, we discovered that PDLIM1 expression was attenuated in AGS/DDP cells; PDLIM1 overexpression boosted AGS/DDP cell apoptosis and elevated the sensitivity of AGS/DDP cells to DDP.

Reportedly, miR-187, a member of the miRNA family, participates in GC development. For instance, miR-187 modulates CRMP1 expression to facilitate GC cell migration and invasion, thus fostering GC development [40]. miR-187 represses FOXA2 to bolster GC growth and metastasis [41]. More of note, miR-187 overexpression dampens the TGF-*β*/Smad signaling pathway to mitigate the resistance of GC cells to DDP [42]. Here, we uncovered that miR-187 expression was heightened in GC; miR-187 overexpression dramatically boosted GC malignant phenotype. Dual luciferase assay revealed that miR-187 targeted PDLIM1, and they were negatively correlated; miR-187 overexpression vigorously impeded PDLIM1 progression. The rescue experiment indicated that PDLIM1 overexpression weakened the procancer function of miR-187 in GC cells.

The Hippo signaling pathway exerts a pivotal function in modulating organ size, migration, and invasion as well as sustaining the balance between cell proliferation and apoptosis [43, 44]. Reportedly, YAP is the primary effector of the Hippo pathway. Dephosphorylated YAP moves into the nucleus, thus promoting gene transcription that modulates proliferation and migration [45]. An increasing amount of evidence has shown that the aberrant activation of YAP incurs the growth-promoting transcription procedure that facilitates cell proliferation, migration, epithelial-mesenchymal transformation, and the stem-cell features of cancer [46]. YAP presents a high expression in GC and other tumors, which bolsters tumor proliferation and metastasis. This is extremely detrimental to the prognosis of cancer patients [47, 48]. For instance, when the nuclear translocation and dephosphorylation of YAP are boosted, GC development also gets promoted [49–51]. Meanwhile, targeting and suppressing YAP and *β*-catenin signaling cramps the malignant behaviors of GC [52]. Here, we discovered that the YAP pathway also partakes in GC progression; PDLIM1 overexpression vigorously represses the mRNA and protein levels of YAP and its target genes AREG, Myc, and CCND2; the levels of nuclear and phosphorylated YAP were lowered in the nucleus. These findings confirmed that PDLIM1 served as a tumor-suppressing factor and hindered the Hippo/YAP signaling pathway in GC.

To conclude, our research has unveiled a novel molecular mechanism for GC treatment: PDLIM1 mediates the Hippo-YAP signaling pathway to exert a cancer-suppressing function and strengthen the sensitivity of GC to cisplatin. Our observation affords significant insights into GC treatment and prognosis. Nevertheless, we have not completely corroborated the reliability of the mechanism *in vivo*, which will be improved in the future.

## Figures and Tables

**Table 1:** The primers used in this study.

Gene name	Primer sequence (5'-3')
PDLIM1	Forward: CCCAGCAGATAGACCTCCAG
	Reverse: GTTGTCTGTGCAGCCTTTGA
miR-187	Forward: TCGTGTCTTGTGTTGCAGC
	Reverse: GTGCAGGGTCCGAGGT
*β*-Actin	Forward: GGCATCCTCACCCTGAAGTA
	Reverse: GAAGGTGTGGTGCCAGATTT

**Table 2:** The correlation between PDLIM1 expression and clinical characteristics in the tissue specimens of GC patients.

Characteristics	Patients	Expression of PDLIM1		*P* value
		High-PDLIM1	Low-PDLIM1	
Total	45	23	22	
Age (years)				0.661
<63	21	10	11	
≥ 63	24	13	11	
Gender				0.668
Male	26	14	12	
Female	19	9	10	
Tumor location				0.837
Bottom	20	10	10	
Body	12	7	5	
Gastric antrum	13	6	7	
Diameter				0.023^∗^
< 3 cm	25	9	16	
≥ 3 cm	20	14	6	
Clinical stage				0.005^∗^
Early	24	17	7	
Middle and late	21	6	15	
Distant metastasis				0.025^∗^
Without	22	15	7	
With	23	8	15	
Vascular invasion				0.023^∗^
Yes	25	9	16	
No	20	14	6	

## Data Availability

The datasets analyzed during the current study are available from the corresponding author on reasonable request.
